# Development of a Carvedilol Oral Liquid Formulation for Paediatric Use

**DOI:** 10.3390/pharmaceutics15092283

**Published:** 2023-09-05

**Authors:** Blanca Chiclana-Rodríguez, Encarnacion Garcia-Montoya, Khadija Rouaz-El Hajoui, Miquel Romero-Obon, Anna Nardi-Ricart, Marc Suñé-Pou, Josep M. Suñé-Negre, Pilar Pérez-Lozano

**Affiliations:** 1 Department of Pharmacy and Pharmaceutical Technology and Physical Chemistry, Faculty of Pharmacy and Food Sciences, University of Barcelona, Av. Joan XXIII, 27-31, 08028 Barcelona, Spain; chiclana.blanca@ub.edu (B.C.-R.); khadijarouaz@ub.edu (K.R.-E.H.); annanardi@ub.edu (A.N.-R.); marcsune@ub.edu (M.S.-P.); jmsune@ub.edu (J.M.S.-N.); perezlo@ub.edu (P.P.-L.); 2 Pharmacotherapy, Pharmacogenetics and Pharmaceutical Technology Research Group, Bellvitge Biomedical Research Institute (IDIBELL), Av. Gran Via de l’Hospitalet, 199-203, 08090 Barcelona, Spain; 3 Quality Assurance Pharmaceutical Sites Director—Laboratorios ALMIRALL, Ctra. de Martorell, 41-61, 08740 Sant Andreu de la Barca, Spain

**Keywords:** carvedilol, paediatrics, liquid formulation, solubility, stability, DoE, cardiovascular disease

## Abstract

Carvedilol (CARV) is an ‘off-label’ β-blocker drug to treat cardiovascular diseases in children. Since CARV is nearly insoluble in water, only CARV solid forms are commercialized. Usually, CARV tablets are manipulated to prepare an extemporaneous liquid formulation for children in hospitals. We studied CARV to improve its aqueous solubility and develop an oral solution. In this study, we assessed the solubility and preliminary stability of CARV in different pH media. Using malic acid as a solubility enhancer had satisfactory results. We studied the chemical, physical, and microbiological stability of 1 mg/mL CARV–malic acid solution. A design of experiment (DoE) was used to optimize the CARV solution’s preparation parameters. A 1 mg/mL CARV solution containing malic acid was stable for up to 12 months at 25 °C and 30 °C and 6 months at 40 °C. An equation associating malic acid with CARV concentrations was obtained using DoE. Microbiological data showed that the use of methylparaben was not necessary for this period of time. We successfully developed an aqueous CARV solution suitable for paediatrics and proven to be stable over a 12-month period.

## 1. Introduction

Carvedilol (CARV) is a non-selective blocker of α- and β-adrenergic receptors used for clinically treating cardiovascular diseases such as hypertension, ischemic heart disease, and congestive heart failure. CARV reduces peripheral vascular resistance via vasodilation, and tachycardia is prevented [1]. CARV has only been authorized for adults, even though several studies report its efficacy in children with heart failure [2].

CARV is as an ‘off-label’ treatment in paediatrics because it is unapproved for this population despite being effective. Over 80% of children with cardiac disease receive ‘off-label’ treatments. Approximately 10% of these children are usually treated with β-blockers, most of them with CARV [3,4]. Clinical trials involving paediatric patients with heart failure have shown a positive impact on left ventricular function, clinical condition, and symptoms of heart failure [5,6,7]. As a result, the European Medicines Agency (EMA) included carvedilol on its list of paediatric and therapeutic requirements for cardiology. This addition is due to the need for an age-appropriate pharmaceutical formulation for treating hypertension and heart failure [8]. CARV has been generally well tolerated in children, with the most commonly reported side effects being dizziness, hypotension, and headaches. For children who experienced a significant decrease in blood pressure at the beginning of CARV therapy, the dose was gradually increased to reach the target dosage. Discontinuation of CARV therapy was implemented for children who experienced severe adverse effects, a situation more prevalent among those aged >10 years and those with a more pronounced initial symptom score [9].

Consequently, moving towards the development of liquid formulations for this patient population is necessary. Children <7 years old are not always able to swallow solid pharmaceutical forms. Moreover, drug dosage is determined by the age and weight of each paediatric patient. The benefits of liquid formulations include dose flexibility and titration, ease of ingestion, and enhanced patient adherence [10]. Similarly, excipients within a paediatric formulation should be chosen appropriately, avoiding potentially toxic or unsuitable excipients. If adding any excipient to a paediatric formulation is deemed necessary, its use and quantity may be justified. Furthermore, this excipient can be added at the lowest concentration where possible [11].

From a pharmaceutical point of view, carvedilol is a racemic compound with low aqueous solubility. According to the current Biopharmaceutics Classification System (BCS), CARV is a BCS Class II compound. Thus, it is permeable and well absorbed after oral administration but is nearly insoluble in water (6–8 μg/mL) [12]. For this reason, all commercialized CARV forms are in a solid state. Its formulation as an oral liquid solution is challenging. CARV compounds contain a secondary amine with a pKa of 7.8. Hence, it exhibits predictably low solubility in neutral or alkaline media, and its increase acidifies the media, reaching a plateau at approximately pH 5. The solubility and dissolution of CARV are strongly dependent on pH. Buffer species significantly influence the solubility and dissolution rate of CARV [13].

CARV has only been commercialized in oral solid dosage form, specifically in tablets. These tablets are manipulated prior to use in hospitals with the aim of improving patient compliance and adherence. To prepare a CARV liquid suspension, CARV tablets are crushed and dispersed in one or more excipients. The most commonly used excipient is a mixture of Ora-Sweet–Ora-Plus (1:1) or Ora-Blend [14,15,16]. Some components of this mixture are not recommended in paediatrics, such as sorbitol, sucrose, and saccharin [10,17]. Moreover, manipulation of CARV tablets, such as crushing or splitting, to formulate a CARV suspension could produce inaccuracies in the dose obtained. Therefore, under- or over-dosing CARV is a risk, a relevant factor for paediatric patients [18].

Another example is SyrSpend SF PH4 (Fagron), a ready-to-use oral liquid vehicle compatible with different APIs (active principal ingredients), including CARV. A small amount of SyrSpend SF PH14 is added to the powder from crushed CARV tablets and mixed to form a CARV suspension [19].

Other liquid formulations of CARV containing unrecommended excipients in paediatrics have been described. In Yamreudeewong et al.’s study [20], CARV tablets were triturated and mixed with deionized water to form a paste. They added 70% sorbitol solution and deionized water to obtain the final volume. However, sorbitol is not recommended for the paediatric population. It has caused different side effects in children, such as gastrointestinal disorders, diabetic complications, and even liver damage [10]. Buontempo et al. prepared a CARV solution containing sorbitol and propylene glycol, among other excipients [21]. Exposure to high doses of propylene glycol may affect the central nervous system, especially in newborns and children <4 years of age. Due to children’s physiological and metabolic immaturity, propylene glycol can accumulate rapidly, causing toxicity [22].

Operto et al. [23] developed two CARV liquid formulations for administration to paediatric and geriatric patients. These formulations contained a high level of PEG-400 (15 and 27% *v*/*v*), as well as suspending agents such as HPMC (hydroxypropyl methylcellulose) (0.25 and 0.50% *w*/*v*). The shelf life of these formulations was 180 days at 4 °C and 180 days at 25 °C, respectively. The PEG-400 maximum recommended daily dose was 10 mg/kg body weight. When newborns and infants are exposed to high doses of PEG-400, gastrointestinal disorders and adverse effects typical of alcoholic solvents may occur [10,24]. Accordingly, PEG-400 may not be a safe excipient for paediatrics.

Approximately 70% of the new molecules developed as APIs are poorly soluble in water. Recent methods for increasing solubility include API and polymer composites obtained by creating amorphous solid dispersions [25], developing nanocarrier systems as nano-slow-release systems using mesoporous materials [26], and generating self-emulsifying systems (SEDDS), which are promising tools to enhance permeation across biological membranes [27].

Many attempts to overcome the low aqueous CARV solubility via encapsulation have been made. CARV encapsulation within nanomicelles could improve drug solubility with commercially available copolymers [28]. Another study developed a CARV nanoemulsion, a solidified self-nanoemulsifying drug delivery system generated with oil and surfactants [29]. CARV nanoparticles have also been developed, one example being CARV-loaded chitosan nanoparticles via the ionic gelation method [30]. Even a liposomal formulation of CARV was prepared using organic solvents [31].

In addition, Khan et al. [32] developed a CARV orally disintegrating mini tablet (ODMT) appropriate for paediatrics. The CARV ODMT doses were 0.5 mg and 2 mg, respectively, since CARV-marketed tablets with the lowest strength were 3.125 mg. Although mini tablets might be suitable for paediatrics, oral solutions remain the preferred choice for more accurate dosing, especially in neonates or children up to 4–5 years old who experience difficulty swallowing solid pharmaceutical forms [10].

The European Union recognised the need for paediatric-centric medicines and introduced the paediatric investigation plan (PIP) to support the authorization of medicines for children [33]. Therefore, the EMA has drafted several guidelines for pharmaceutical development and clinical trials for paediatric products [11,34,35] to assist in the development of paediatric formulations.

Thus, developing a liquid formulation is a good alternative to administering CARV to the paediatric population. The most preferred dosage form is a solution since it is easy to fractionate for paediatric doses while ensuring the right API content in each dose is administered [35]. Furthermore, this formulation should contain excipients approved for paediatric patients. CARV doses prescribed for paediatrics in infants and children <12 years of age are 0.05–0.10 mg/kg (child’s weight)/12 h at the beginning of treatment. A maximum dose of 25 mg/12 h can be administered if the patient tolerates the drug well [36].

Our aim for the present study was to formulate and characterise a paediatric-appropriate CARV oral solution, which has not been developed up until now. CARV concentration should be 1 mg/mL when calculating the dose according to the paediatric patient’s body weight, since CARV dose depends on this. Additionally, most paediatric syringes for dose administering are graduated on a decimal scale. Moreover, this formulation should be prepared from CARV raw material, not from tablets.

Hence, we sought to develop an aqueous liquid vehicle containing excipients appropriate for paediatrics, in which CARV was soluble at a concentration of 1 mg/mL. This formulation should remain stable for at least 15 days at 25 °C if it does not contain preservative agents and up to 6 months at 25 °C if it contains preservative agents. However, if the formulation without preservative agents performs well in the efficacy test for antimicrobial preservation of oral preparations (Challenge Test), it could be preserved for up to 6 months. These dates are the sell-by dates for most liquid compounded medications formulated in hospitals [37].

## 2. Materials and Methods

### 2.1. Materials

#### 2.1.1. Pharmaceutical Development Chemicals

*European Pharmacopoeia*-grade (Ph. Eur.) carvedilol was kindly donated by Moehs (Barcelona, Spain). Ph. Eur.-grade malic acid-DL was purchased from FAGRON IBERICA (Barcelona, Spain). Other materials included dihydrogen potassium phosphate, sodium acetate, and citric acid monohydrate from Merk (Barcelona, Spain). We also obtained glacial acetic acid, hydrochloric acid, ortho-phosphoric acid, and sodium hydroxide from Panreac Applichem (Barcelona, Spain).

The water used for analysis was MilliQ grade. All solvents used were analytical grade.

#### 2.1.2. Microbiological Test Materials

Methylparaben was purchased from FAGRON IBERICA (Barcelona, Spain) and PEG-400 from Thermo Fisher Scientific (Barcelona, Spain).

Trypto-Casein Soy Agar (TSA) (Oxoid, Madrid, Spain) and Sabouraud Dextrose Agar (Oxoid, Madrid, Spain) were used as culture media. We used sodium chloride-peptone buffer at pH 7.7 for sample neutralising and as a sterile suspending fluid (Scharlau, Barcelona, Spain). Beerens Diluent 3% was used to neutralise parabens.

### 2.2. Solubility Studies

#### 2.2.1. Determination of CARV Solubility at Different pH Levels

Firstly, we studied the solubility of CARV at pH 6.8 with phosphate buffer (potassium dihydrogen phosphate and sodium hydroxide), pH 4.5 acetate buffer (sodium acetate and acetic acid), and pH 1.2 (hydrochloric acid and sodium chloride) using the shake flask method, a saturation method [38]. The preparation for these media is described in the *European Pharmacopoeia*’s *Recommendations on dissolution testing* [39].

To do so, we added an excess amount of CARV in a solid state to a glass containing the solvent until system saturation at 25 °C for up to 24 h. The mixture was homogenized with a magnetic stirrer. Afterwards, the sample was filtered through a 0.45 µm PVDF membrane filter (Agilent, Barcelona, Spain). An aliquot of this filtrate was collected in 2 mL HPLC vials to be tested. Samples were quantified using an HPLC (high-performance liquid chromatography) method described in this section. Solubility was calculated as the mean of three replicates.

Chromatographic separation of CARV was performed using a Luna C18 column 150 mm × 46 mm, id 5 μm, manufactured in stainless steel (Phenomenex, Barcelona, Spain). The mobile phase consisted of HPLC-quality acetonitrile and buffer solution (pH 4.5, potassium dihydrogen phosphate), and an isocratic program was used (35:65, respectively). The flow rate was 1 mL/min. The DAD detector was operated at 240 nm. The injection volume was 5 μL. HPLC analysis was conducted at 40 °C. Each determination required 30 min. This method is based on the one described in the *European Pharmacopoeia* 11th edition with some modifications [40]. CARV at 1 mg/mL was the working concentration of the target formula.

#### 2.2.2. Determination of CARV Apparent Solubility at 1 mg/mL in Acid Media

We studied CARV’s apparent solubility at 1 mg/mL at different acid media and pH, using the shake flask method. pH media between 2.0 and 5.0 were studied. These acid media were acetic media (acetic acid and sodium acetate), phosphoric media (potassium dihydrogen phosphate and phosphoric acid), citric media (citric acid monohydrate and sodium hydroxide), and malic media (malic acid). Firstly, each medium was prepared; then, a sufficient amount of CARV was added to each medium at 1 mg/mL. CARV was mixed with a magnetic stirrer at 25 °C for up to 24 h. Afterwards, we studied the apparent solubility and stability of CARV.

We also studied CARV saturation in the media, in which CARV was more soluble. We performed a saturation test using the shake flask method until system saturation at 25 °C. CARV solubility was calculated as the mean of three replicates.

### 2.3. Stability Studies

#### 2.3.1. Preliminary Stability Study

We studied the stability of 1 mg/mL of CARV formulations in acetic (F1 and F2), citric (F3 and F4), and malic acid media (F5 and F6) at 25 °C and/or 40 °C for 12 weeks. CARV formulations of 1 mg/mL, F1 to F6, included in this preliminary stability study are represented below:

- F1 and F2 formulations: 0.5 g/100 mL of sodium acetate trihydrate and 0.17 g/100 mL of acetic acid for F2 and additional acetic acid up to pH 4.0 for F1;
- F3 and F4 formulations: 0.8 g/100 mL of sodium hydroxide and 10.0 g/100 mL of citric acid monohydrate for F4 and additional citric acid up to pH 2.0 for F3;
- F5 and F6 formulations: 1.7 g/100 mL of malic acid for F5 and 0.8 g/100 mL of malic acid for F6.

To prepare each medium, we weighed, transferred, and dissolved each component into a beaker containing purified water up to a certain pH value. A sufficient amount of CARV was added to each medium at 1 mg/mL and mixed via magnetic stirring until complete dissolution. After each formulation was prepared, every batch was split into 20 mL capped glass vials. Vials were protected from light.

Samples were collected at different times for 12 weeks and tested for pH (pH meter, HANNA Instruments, Guipúzcoa, Spain), appearance, and CARV assay. We evaluated their appearance to verify homogeneity and no presence of precipitation. Using the validated HPLC method described in this section (results given as mean ± SD), the CARV assay percentage was also evaluated as a parameter considering the initial time. Samples were tested in duplicate at each time of analysis. The specifications for each parameter were:

- Appearance: clear solution, translucent, without undissolved particles;
- pH: initial pH ± 0.2;
- CARV assay (%): 95–105.

#### 2.3.2. Final Stability Study

We studied the stability of the chosen formulation after finishing the preliminary stability test at different conditions for 12 months. We prepared three batches of this formulation, packaged them in 30 mL amber bottles, and stored them at 25 °C, 30 °C (for 12 months), and 40 °C (for 6 months). CARV degradation occurs when CARV is exposed to ambient light in a solution state. Therefore, we stored CARV formulations in amber-capped bottles.

Samples of each batch were collected at different times (1, 3, 6, 9, and 12 months) and subsequently evaluated in duplicate following the International Conference on Harmonization (ICH) guidelines [41]. Evaluation parameters and their specifications are the same as described in the preliminary stability study.

Appearance and pH were checked with samples obtained directly from the bottle. However, before testing the CARV assay, every sample was filtered through a syringe filter with a pore size of 0.45 µm (Agilent, Barcelona, Spain) and diluted with the mobile phase at 1/10 dilution to quantify CARV content via HPLC.

### 2.4. Design of Experiment (DoE)—Optimization of Preparation of CARV Solution

Pharmaceutical products must be developed through proper planning to avoid failure and ensure quality. According to this principle, a method of quality by design (QbD) was created and described in the guidelines of the International Conference on Harmonization (ICH Q8 R2). A statistical method known as DoE is used to mathematically describe the relationships between tested components, their interactions, and product quality. DoE plans allow one to optimize these parameters and precisely identify existing interactions.

Given the desired quality of CARV formulations, it may be possible to identify relationships between the examined factors and response values, thereby creating a design space and finding optimal conditions for the CARV solution’s preparation process. DoE tools such as Minitab 21.0 (Minitab, LLC, State College, PA 16801 USA) can help optimize the formulating process of CARV solutions.

Therefore, in this study, we prepared CARV solutions using certain acids as acidifying and solubilizing agents with the help of a DoE program with a full factorial 3^2^ design (meaning 2 factors at 3 levels). We used a 3^2^ experimental design to optimize the CARV solution’s preparation parameters.

We considered CARV concentration and acid concentration, in which CARV was more soluble, as independent variables (factors). We developed three runs with three levels: minimum, medium, and maximum ranges of CARV and acid concentrations.

The pH value and CARV assay were regarded as dependent variables. We selected the optimum levels of these dependent variables based on the obtained results. Proper optimization was helpful in preventing CARV precipitation, considering that CARV is not soluble at certain pH values.

Using statistical design (response surface methodology), an equation was designed to predict responses based on significant factor levels.

### 2.5. Efficacy of Antimicrobial Preservation (Challenge Test)

#### 2.5.1. Preparation of CARV Solutions

We prepared and tested three 5 mg/mL CARV solutions in acidic medium for microbiological stability in the presence and absence of methylparaben (methyl 4-hydroxybenzoate), a preservative agent. Methylparaben was chosen for its suitability to paediatrics [10].

The first formula contained 0.2% methylparaben, the second formula contained 0.1% methylparaben, and the third did not have any preservative agents. Firstly, methylparaben had to be dissolved in polyethylene glycol-400 (PEG-400) at 10.0%. Afterwards, a mixture of methylparaben and PEG-400 was added to 5 mg/mL CARV solutions in acid medium to prepare the first and second formulas. We distributed test CARV solutions in 100 mL sterile amber glass bottles and stored them at room temperature.

#### 2.5.2. Description of the Test

We determined the efficacy of antimicrobial preservation based on the 11.0 *European Pharmacopeia* monograph 5.1.3 [42]. The test consists of challenging the preparation with a prescribed inoculum of suitable micro-organisms, storing the inoculated preparation at a prescribed temperature, withdrawing samples from the container at specified intervals of time, and counting the organisms in the withdrawn samples.

The preservative properties of the preparation are adequate if, under the test conditions, there is a significant fall or no increase in the number of micro-organisms in the inoculated preparation at the times and temperatures prescribed.

The criteria for evaluating antimicrobial activity are expressed as a log_10_ reduction in the number of viable micro-organisms compared to the inoculum value. For oral preparations, the log_10_ reduction must be ≥3 for bacteria and ≥1 for fungi inocula after 14 days. No increase was detected in the number of viable micro-organisms compared to the previous reading for bacteria and fungi inocula after 28 days.

#### 2.5.3. Test Conditions

Samples were distributed in aliquots of 10 mL and placed in sterile containers. These aliquots were inoculated with 100 µL of the microbiological suspension. The inoculated culture media included *P. aeruginosa*, *S. aureus*, *E. coli* (bacteria), *C. albicans*, and *A. brasiliensis* (fungi).

We determined the number of viable micro-organisms by plate count at prescribed times. We ensured that specific inactivators eliminated the product’s residual antimicrobial activity.

## 3. Results

### 3.1. Solubility

Table 1 presents the solubility data for CARV in three pH values (1.2, 4.5, and 6.8) at 25 °C. The aim of our research was to solubilize CARV at a concentration of 1 mg/mL. Table 1 shows that the CARV solubility at pH 1.2 and 6.8 is extremely low; therefore, CARV at 1 mg/mL is only soluble in a pH 4.5 acetate buffer. These results coincide with CARV’s low solubility data in the aforementioned neutral and alkaline media [12]. CARV solubility increased in acidic media (acetate buffer pH 4.5). However, CARV’s solubility decreased again at pH < 2 as hydrochloric acid media pH 1.2.

Accordingly, we studied CARV’s apparent solubility at 1 mg/mL in different acid media and pH. Our results are shown in Table 2. pH values from 2.0 to 5.0 were tested. pH values <2.0 were not tested because they were considered too acidic for a paediatric oral solution [43,44]. CARV is not soluble at pH > 5.0, as shown in Table 1 and Table 2.

CARV is soluble in phosphoric media at pH 2.0 and 2.5, but precipitation was observed after 24 h. Consequently, phosphoric media were discarded as a solvent.

CARV is soluble in acetic media at pH 3.5, but its dissolution is difficult. It required too much time and high speeds for its dissolution. CARV 1 mg/mL is soluble and stable after 24 h in acetic media pH 4.0–4.5, citric media pH 2.0–2.5, and malic media pH 2.0–3.0. Therefore, we studied CARV stability in these solvents for three months at different conditions (Table 3, Table 4 and Table 5).

### 3.2. Preliminary CARV Solutions

#### Stability

We evaluated the stability of 1 mg/mL CARV solutions from F1 to F6 in different acid media for 12 weeks under different temperature conditions.

At each sampling time, we maintained the visual appearance of all formulations (F1 to F6) throughout the entire study. No precipitation of CARV occurred in any formulation included in the stability study. Solutions were considered stable if no precipitation occurred and the mean drug concentration was found within the range of 95–105% of the labelled concentration.

No changes in pH more than ±0.2 occurred in any formulation (F1 to F6) after 12 weeks for all conditions studied.

Table 3, Table 4 and Table 5 show the results of the CARV assay percentage in formulations F1–F6. The initial CARV concentration was 100%. CARV assay in acetic acid media (F1 and F2) and malic acid media (F5 and F6) at 25 and 40 °C stayed above 100.0% during the 12 weeks of the study. These data were verified with the subsequent final stability study in Section 3.3.3.

When CARV was in citric acid media, F3 and F4 were the only formulations in which the CARV assay percentage decreased during the study. CARV assays of F3 and F4 at 12 weeks were above 95.0%. Although CARV assays were higher than the 95.0% specification in F3 and F4 after 12 weeks at room temperature, the CARV content was unstable due to its reduction in those weeks. These losses in CARV content, with respect to the initial time, indicate poor CARV stability in citric acid media. For this reason, we discontinued studying CARV 1 mg/mL in citric acid media.

According to the results of the F1, F2, F5, and F6 CARV assays presented in Table 3 and Table 5, which had good pH stability and a consistent appearance, we could conclude that CARV is more stable in acetic and malic acid media than in citric media.

Although CARV at 1 mg/mL in acetic acid media is soluble and stable at studied conditions, acetic acid is nauseating and should not be used to compound paediatric oral solutions [45]. Adequate palatability plays an important role in patient acceptability, especially in oral liquid formulations [11]. We did not continue using acetic acid medium as a solvent for these reasons. Ultimately, malic acid medium was the chosen solvent for CARV. We will continue studying CARV at 1 mg/mL in malic acid media.

Moreover, pH adjustment is also important in pharmaceutical development. The optimum pH for an oral solution is neutral to slightly acidic, and pH values down to 3.0 are acceptable only when the solution lacks buffer capacity [46]. A higher pH value in which CARV is soluble using malic acid is 3.0. By using malic acid at pH 2.7, the formulation aims to maintain the solubility of CARV and prevent it from precipitating out of the solution. Hence, malic acid at pH 2.7 was considered the most suitable solvent for formulating a 1 mg/mL CARV solution. We studied this formulation’s 12-month stability and optimized its preparation.

### 3.3. Malic Acid CARV Formulations

#### 3.3.1. Saturation Study

Since malic acid at pH 2.7 is the most suitable solvent for CARV solution, we studied the saturation concentration of CARV in this medium. The solubility data and saturation concentration for CARV in pH 2.7 malic acid medium at 25 °C were 6.550 (±0.200) mg/mL (*n* = 3).

#### 3.3.2. Design of Experiment (DoE)

Regarding the saturation concentration of CARV in malic acid medium, we proposed optimizing the preparation of CARV solutions at 1, 3, and 5 mg/mL in this medium using a DoE.

Although we aimed to perform a CARV solution of 1 mg/mL, the selection of CARV strengths was based on individual child weights. We also studied 3 and 5 mg/mL CARV solutions. Our objective was to administer the lowest volume of CARV solution possible.

CARV formulations included in the DoE study were coded as F7. Nine formulations were studied, F7_1–F7_9, as a full factorial 3^2^ experimental design. We formulated formulations in three runs and prepared 27 batches in total. Table 6 shows the composition of F7_1–F7_9 formulations. Table 7 and Table 8 show DoE study results for CARV assay, pH stability, and appearance after 24 h of F7_1–F7_9 formulations.

**Figure 1:**
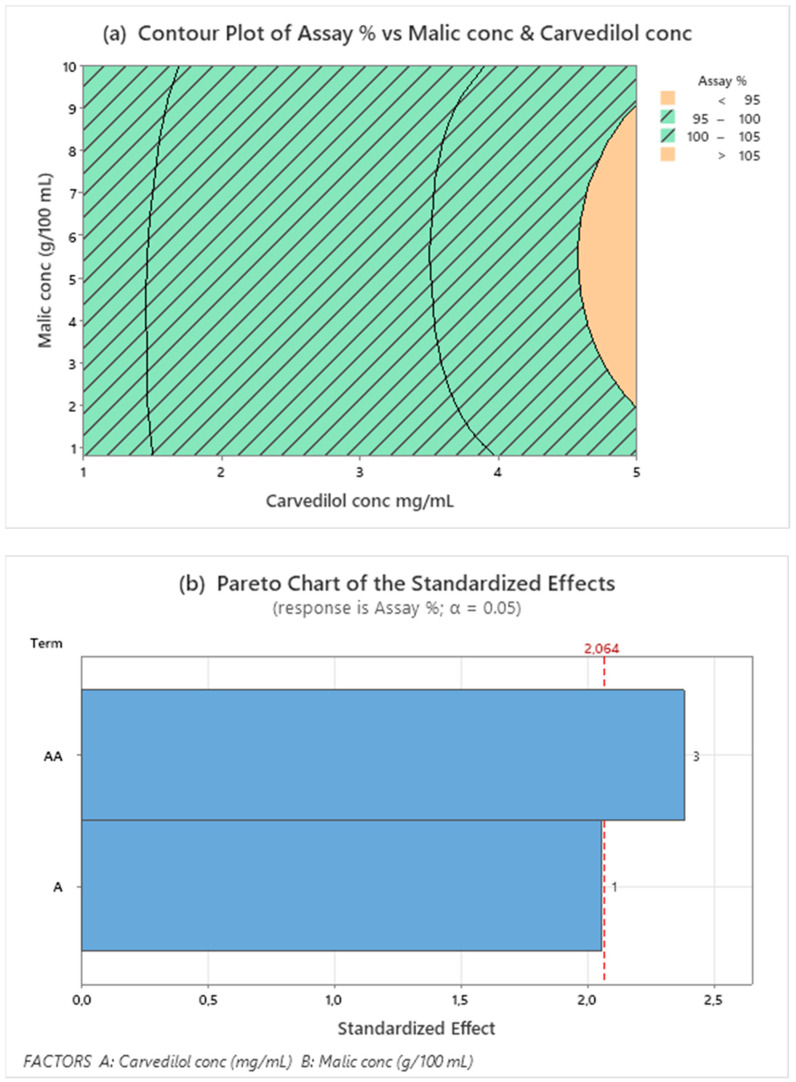
Contour plot for assay (**a**) response values using malic acid and CARV concentrations as predictor variables. The striped area shows the optimal working range for both responses. Pareto’s diagram for assay (**b**) includes factors with a statistical significance of α = 0.05.

**Figure 2:**
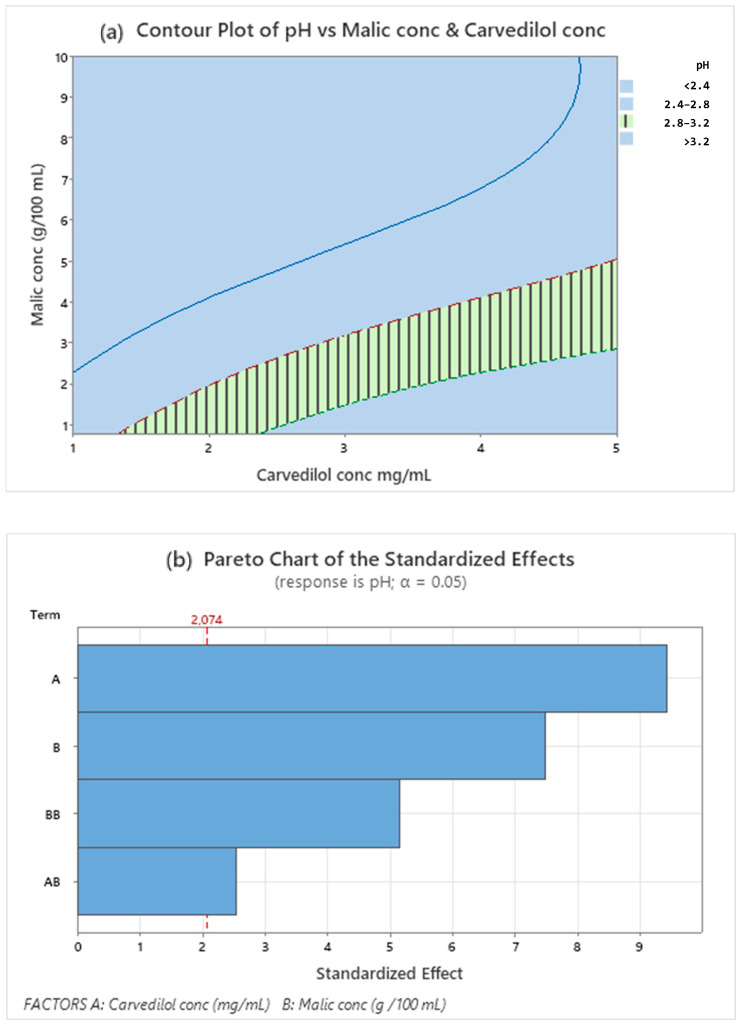
Contour plot for pH (**a**) response values using malic acid and CARV concentrations as predictor variables. The striped area shows the optimal working range for both responses. Pareto’s diagram pH (**b**) includes factors with a statistical significance of α = 0.05.

The regression analysis of DOE data provided by Minitab includes significant response factors: pH and CARV assay (%), as shown in the Pareto charts in Figure 1b and Figure 2b. Factors exceeding the standard line of 95.0% indicate factors that significantly influenced the response studied. Figure 1b shows that only CARV concentration factors at orders 1 and 2 (quadratic terms) significantly influenced the CARV assay response. The contour plot of the CARV assay shows an interesting area delimited by the response value between 95.0 and 105.0%. Therefore, we verified that the whole area was suitable. Figure 1 and Figure 2 represent the optimum working area in oblique (Figure 1a) and vertical lines (Figure 2a).

The quadratic term of malic acid had no significant influence (0.260 *p*-value), and malic acid concentration alone has some influence (0.051 *p*-value) on the CARV assay within the studied range. Since this equation does not explain the model for this response, continuing studies with a larger range of CARV and malic acid concentrations is necessary to define a design space for these concentrations.

Regarding the pH response, the interval studied indicates that the factors are significant and should be optimized (Figure 2a,b). As shown in the Pareto chart (Figure 2b), both malic acid and CARV concentrations and their interactions significantly influence pH response.

The recommended working range for proper pH value, around 3.0, corresponds to the remarked vertical line zone in the contour plot (Figure 2a): malic acid concentration depends on the desired CARV concentration. This finding is also presented in the regression equation (Equation (1)), and the R-sq (adjustment) is 95.2%. All equation coefficients have a < 0.05 *p*-value; thus, they are statistically significant. Therefore, this model is suitable for predicting combinations of different factor levels within the studied framework.

Minitab outcome for the regression equation of pH as response values using CARV and malic acid concentrations.

(1) $$\mathrm{pH}=2.665+0.2643\;\mathrm{CC}\;-0.2384\;\mathrm{MAC}\;+0.01352{\;\mathrm{MAC}}^{2}-0.01087\;\mathrm{CC}\times \mathrm{MAC}$$

Abbreviations: CC = CARV concentration (mg/mL); MAC = malic acid concentration (g/100 mL).

**Figure 3:**
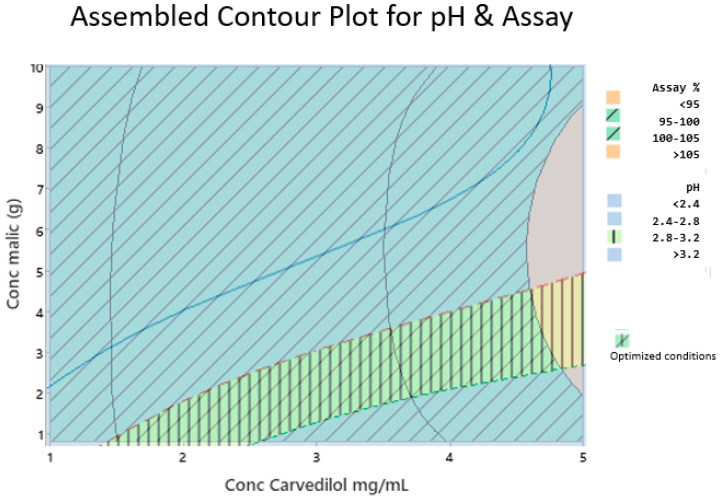
Assembled contour plot for pH and CARV assay (%). Compliance with specifications for the CARV assay percentage (95.0–105.0%) are represented by oblique lines. Compliance with specifications for pH (2.8–3.2) are represented in vertical lines.

Given the desirability of the CARV solution, we could identify relationships between the examined factors and response values, thereby creating a design space and finding optimal conditions to prepare the formulation. We assembled the contour plot of the CARV assay and pH areas to obtain an optimal design space (Figure 3). In Figure 3, optimal conditions are shown at the intersection of specifications, oblique and vertical lines, pH (2.8 to 3.2), and CARV assay (95.0–105.0%).

We conducted a DoE to optimize the two critical parameters of the final formula. One critical parameter is pH since it must be suitable for paediatrics. pH amounts <2.0 are not appropriate for them [43,44]. The other critical parameter is the CARV assay, which ensures the correct dose. Our DoE study results verified that malic acid concentration did not affect the CARV assay response within the CARV concentration range studied (1.0 to 5.0 mg/mL). Working with a CARV concentration >5.0 mg/mL may be necessary; however, more assays would be required. By contrast, malic acid and CARV concentrations significantly affected the pH of the formula, and both concentrations interacted with each other (Figure 3).

#### 3.3.3. Stability of Final CARV Formulations

We included the F7_1_1 formulation in the stability study and prepared three batches. Every batch was analysed in duplicate at each stability time. The poolability of results from the three batches, batch × time interaction, was previously verified with a significance level of 0.477. Since the *p*-value is >0.25, the results data from the three batches can be combined for a single shelf life.

**Figure 4:**
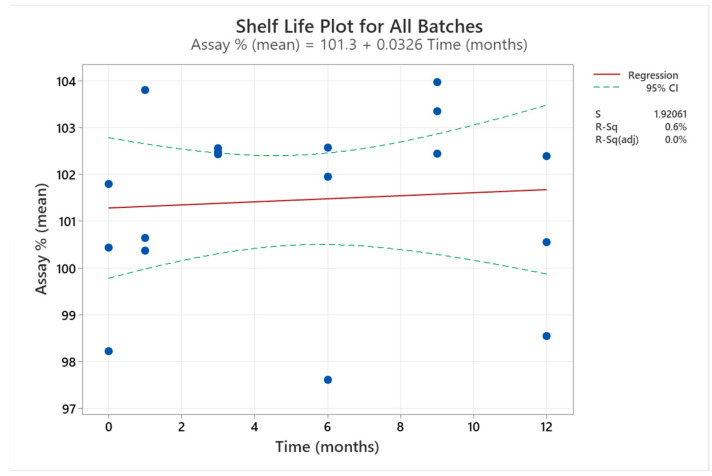
Shelf-life plot for the mean of F7_1_1 three batches included in the final stability study. The results of the three batches at each time are represented by the bule dots in the graph.

Table 9 presents CARV assay results for F7_1_1. All results were according to the 95.0–105.0% specification. The slope of these results did not significantly differ from the horizontal line (Figure 4).

The initial F7_1_1 pH value was 3.0, and initial data on F7_1_1 appearance indicated that it was a clear, translucent solution without any undissolved particles. pH results for F7_1_1 were highly stable over time with a range of variation ±0.05. They are not represented for these reasons. F7_1_1 appearance conformed and kept to the specifications at each studied time. We can conclude that 1 mg/mL CARV aqueous formulation in malic acid has good stability according to the CARV assay (%), pH, and appearance results for 12 months.

We analysed the CARV assay results (%) for three stability batches of F7_1_1 for 12 months using Minitab 21.0 to extrapolate the shelf life of the formulation (Figure 4). The correlation between CARV assay (%) and time is a straight line with a zero slope (97.5% adjustment), indicating no significant variation in CARV assay (%) during the study. However, these data were used to extrapolate 24 months of stability time from ICH Q1(a). All data accomplished the upper and lower specifications.

#### 3.3.4. Efficacy of Antimicrobial Preservation (Challenge Test)

We coded CARV 5 mg/mL formulations as F8 in the Challenge Test. F8_1 contained 0.2% methylparaben, F8_2 contained 0.1% methylparaben, and F8_3 contained no preservative agents.

Table 10 shows the efficacy results of antimicrobial preservation from F8_1 to F8_3. Results are expressed as a log_10_ reduction in the number of colony-forming units per gram (ufc/g). The log_10_ reduction is ≥3 for bacteria and ≥1 for fungi inocula after 14 days from F8_1 to F8_3. No increase was detected in the number of viable micro-organisms in any formulation compared to the previous reading after 28 days for bacteria and fungi inocula.

According to the microbiological stability assessment comparing CARV 5 mg/mL solutions with (F8_1 and F8_2) and without preservatives (F8_3), all formulations complied with the *European Pharmacopoeia*’s specifications for antimicrobial preservation in oral preparations under the evaluated conditions.

Despite methylparaben being the preservative agent most suitable for paediatrics, the EMA recommends avoiding preservatives wherever possible in the case of paediatric formulations. Preserving a paediatric preparation and choosing a preservative system at the lowest concentration feasible should be justified in terms of a benefit–risk balance [11].

## 4. Discussion

We achieved the objective proposed in this paper, namely the development of a stable oral liquid formulation for use in paediatrics, through our experiments. Different studies were conducted to ensure the quality of the proposed formula: solubilization studies, pre-stability studies, optimization of its preparation (DoE), microbiological stability studies, and stability studies. Solubility and pre-stability studies allowed the selection of acid malic as the best acid to solubilize CARV. Considering the consulted literature, this is a novel proposal and represents an important advance in administering CARV to children.

Some CARV solutions for paediatrics were developed by Operto et al. However, these solutions contain a high percentage of PEG-400 co-solvent, which is not appropriate for paediatrics. Furthermore, the shelf life of the formulation with the best stability is 180 days at 25 °C [23]. The CARV solution for paediatrics described in our research was stable for 12 months at 25 and 30 °C and 6 months at 40 °C, which is a significant improvement in terms of shelf life.

CARV suspensions for use in paediatrics have been developed [14,15,16,19,21] because their availability is essential in treating cardiovascular diseases in children [8]. Furthermore, most of these suspensions are fabricated from CARV tablets. However, if taste and drug release characteristics are appropriate, solutions are preferred over suspensions due to better oral acceptance. In addition, instructions on shaking the product to ensure correct dosing are necessary for suspensions [35].

Hamed et al. [13] concluded that the solubility and dissolution rate of CARV were clearly dependent on pH and buffer species of the dissolution media. Thus, CARV solubility was investigated in different media at different pH levels. Our results coincided with CARV’s low solubility data in neutral and alkaline media [12]. CARV had high solubility and stable results in acidic media, similar to acetic and malic media. Moreover, the basicity of the aliphatic NH (pKa 7.8) accounts for its high solubility in these media.

Improving CARV’s water solubility with malic media as an acidifying agent has never been described. CARV’s high solubility in an acetate buffer (pH 4.5) has already been reported. Using acetic buffer as a solvent, CARV is in its protonated form at 99.95%. The protonated base forms a water-soluble salt with the anionic form of acetic acid, resulting in increased dissolution [13]. Depending on the buffer species, CARV is soluble in different pH media, notably at pH 3.5–4.5 in acetic media and pH 2.0–3.0 in malic media.

CARV at 1 mg/mL, the target concentration, is soluble in malic media at pH 2.5–3.0 and stable up to 12 months at 25 and 30 °C. Even though CARV’s solubility in this media at 3 and 5 mg/mL was investigated using a DoE, this research was not continued due to a lack of time. CARV’s stability in malic media at 3 and 5 mg/mL should be studied in further studies.

The proposed CARV oral liquid formulation does not contain any unrecommended excipients for paediatrics. Malic acid is listed in the GRAS (Generally Recognized as Safe) list from FDA (U.S. Food and Drug Administration, Silver Spring, MD, USA), and both isomers are accepted as food additives to oral preparations in Europe. Malic acid is widely used in pharmaceutical oral formulations as an acidulant and flavouring agent due to its slight apple flavour. Moreover, it has antioxidant properties [47]. No data about its toxicity in paediatrics have been exposed [48].

Despite being an oral aqueous formulation, adding any preservative agents to the CARV solution is not necessary owing to Challenge Test results. This finding may be due to the formulation’s characteristics such as a low pH value. Accordingly, the final CARV formulation had a preservative-free composition.

Palatability (appreciation of smell, taste, aftertaste, and texture) is a main element of paediatric patients’ acceptability of an oral liquid formulation. Hence, flavours and sweeteners may be necessary to achieve this goal [11]. The next step in this research is to optimize the qualitative and quantitative composition, including added flavouring and sweetening agents to improve the formula’s acceptability. Validating the CARV analysis method using the optimized CARV formulation is another step that must be performed. Furthermore, a stability study following the ICH guidelines was performed to verify the physicochemical, formulation-related, and microbiological parameters obtained in this preliminary study.

## 5. Conclusions

We developed an oral CARV liquid formulation containing excipients suitable for paediatrics. We used a 1 mg/mL CARV solution, which was chemically, physically, and microbiologically stable for 12 months at 25 °C and 30 °C, as well as 6 months at 40 °C. From our design of experiment results, we concluded that the final pH of the formula depends on CARV and malic acid concentrations. However, these factors do not significantly influence the CARV assay percentage. Using Equation (1), the formulator should check previously recommended optimal malic acid concentrations for certain CARV concentrations to ensure compliance with the specifications. Another advantage of this research is that adding preservative agents to the formula for 12-month validity is not necessary.

## Figures and Tables

**Table 1:** Carvedilol solubility values (mg/mL) at three pH values. Data presented as mean ± SD; *n* = 3.

pH	Solubility (mg/mL)
1.2	0.027 (±0.003)
4.5	1.280 (±0.050)
6.8	0.037 (±0.002)

**Table 2:** Carvedilol solubility at 1 mg/mL in different pH media.

pH	Acetic Media	Phosphoric Media	Citric Media	Malic Media
2.0	NS	S/not stable > 24 h	S/stable > 24 h	S/stable > 24 h
2.5	NS	S/not stable > 24 h	S/stable > 24 h	S/stable > 24 h
3.0	NS	NS	NS	S/stable > 24 h
3.5	S/difficult dissolution	NS	NS	NS
4.0	S/stable > 24 h	NS	NS	NS
4.5	S/stable > 24 h	NS	NS	NS
5.0	NS	NS	NS	NS

Abbreviations: S = soluble; NS = not soluble.

**Table 3:** Preliminary stability results. CARV assay stability results for F1 and F2 formulations (CARV in acetic media) at different temperatures for 12 weeks. Mean ± SD (*n* = 2).

	CARV Assay (%)				
Storage Temperature	1 Week	2 Weeks	4 Weeks	8 Weeks	12 Weeks
F1 formulation (acetic media pH 4.0)					
25 °C	103.07 ± 1	102.70 ± 2	102.50 ± 1	101.80 ± 1	101.78 ± 2
40 °C	-	102.20 ± 3	101.07 ± 1	-	101.90 ± 1
F2 formulation (acetic media pH 4.5)					
25 °C	101.67 ± 1	101.37 ± 1	101.24 ± 1	101.16 ± 1	101.10 ± 1
30 °C	101.26 ± 2	101.60 ± 1	101.45 ± 3	101.54 ± 2	101.50 ± 1
40 °C	101.05 ± 1	100.50 ± 2	100.10 ± 2	-	-

**Table 4:** Preliminary stability results. CARV assay stability results for F3 and F4 formulations (CARV in citric media) at different temperatures for 12 weeks. Mean ± SD (*n* = 2).

	CARV Assay (%)				
Storage Temperature	1 Week	2 Weeks	4 Weeks	8 Weeks	12 Weeks
F3 formulation (citric media pH 2.0)					
25 °C	98.13 ± 2	97.02 ± 1	96.38 ± 1	96.40 ± 2	95.02 ± 1
F4 formulation (citric media pH 2.5)					
25 °C	100.46 ± 1	98.26 ± 3	97.02 ± 2	96.49 ± 1	95.78 ± 1

**Table 5:** Preliminary stability results. CARV assay stability results for F5 and F6 formulations (CARV in malic media) at different temperatures for 12 weeks. Mean ± SD (*n* = 2).

	CARV Assay (%)				
Storage Temperature	1 Week	2 Weeks	4 Weeks	8 Weeks	12 Weeks
F5 formulation (malic media pH 2.0)					
25 °C	103.01 ± 1	102.26 ± 3	103.61 ± 2	101.50 ± 1	101.87 ± 2
40 °C	-	-	102.50 ± 1	101.04 ± 1	101.10 ± 1
F6 formulation (malic media pH 2.7)					
25 °C	102.40 ± 1	102.20 ± 1	102.50 ± 1	101.30 ± 2	101.20 ± 1
40 °C	-	-	102.05 ± 1	101.70 ± 1	100.80 ± 1

**Table 6:** Composition of CARV formulations included in the DoE study.

	F7_1	F7_2	F7_3	F7_4	F7_5	F7_6	F7_7	F7_8	F7_9
CARV (mg/mL)	1			3			5		
Malic acid (% *m*/*v*)	0.8	5.4	10.0	0.8	5.4	10.0	0.8	5.4	10.0

**Table 7:** CARV assay results for F7_1–F7_9 formulations and their batches included in the DoE study. Mean (*n* = 2).

		Malic Acid Concentration					
		0.8%		5.4%		10.0%	
		Batch	CARV Assay (%)	Batch	CARV Assay (%)	Batch	CARV Assay (%)
CARV concentration (mg/mL)	1	F7_1_1	102.14	F7_2_1	100.73	F7_3_1	102.67
		F7_1_2	101.31	F7_2_2	102.66	F7_3_2	103
		F7_1_3	100.12	F7_2_3	101.78	F7_3_3	99.76
	3	F7_4_1	99.07	F7_5_1	99.53	F7_6_1	97.86
		F7_4_2	100.36	F7_5_2	100.11	F7_6_2	99.23
		F7_4_3	97.09	F7_5_3	96.88	F7_6_3	99.68
	5	F7_7_1	112.38	F7_8_1	99.33	F7_9_1	112.73
		F7_7_2	98.44	F7_8_2	112.65	F7_9_2	100.35
		F7_7_3	97.66	F7_8_3	111.93	F7_9_3	96.78

**Table 8:** pH and appearance results for F7_1–F7_9 formulations and their batches included in the DoE study. Mean (*n* = 2).

		Malic Acid Concentration								
		0.8%			5.4%			10.0%		
		Batch	pH	Appearance after 24 h	Batch	pH	Appearance after 24 h	Batch	pH	Appearance after 24 h
CARV concentration (mg/mL)	1	F7_1_1	2.6	Conform	F7_2_1	2.0	Conform	F7_3_1	1.80	Conform
		F7_1_2	2.6		F7_2_2	2.0		F7_3_2	1.80	
		F7_1_3	2.75		F7_2_3	2.05		F7_3_3	1.85	
	3	F7_4_1	3.2	Not conform	F7_5_1	2.35	Conform	F7_6_1	1.85	Conform
		F7_4_2	3.4		F7_5_2	2.35		F7_6_2	2.0	
		F7_4_3	3.55		F7_5_3	2.50		F7_6_3	2.15	
	5	F7_7_1	3.6	Not conform	F7_8_1	2.75	Conform	F7_9_1	2.40	Conform
		F7_7_2	3.6		F7_8_2	2.65		F7_9_2	2.45	
		F7_7_3	3.95		F7_8_3	2.85		F7_9_3	2.6	

**Table 9:** Stability CARV assay data for F7_1_1 at different temperatures for 12 months.

	F7_1_1 Formulation					
Storage Temperature	T0	1 Month	3 Months	6 Months	9 Months	12 Months
	% CARV Assay (mean of three batches ± SD)					
25 °C	100.16 ± 1.48	101.60 ± 1.55	102.49 ± 0.05	100.71 ± 2.20	103.25 ± 0.63	100.50 ± 1.57
30 °C		102.71 ± 0.84	102.02 ± 0.58	100.37 ± 1.16	103.56 ± 0.62	102.63 ± 1.62
40 °C		101.96 ± 1.46	102.03 ± 0.77	102.20 ± 1.10	-	-

**Table 10:** Results of Challenge Test in F8_1 to F8_3. Data presented as log_10_ reduction in different culture media at 0 h, day 14, and 28.

		*P. aeruginosa* (ATCC 9027)	*S. aureus* (ATCC 6538)	*E. coli* (ATCC 8739)	*C. albicans* (ATCC 10231)	*A. brasiliensis* (ATCC 16404)
Inoculum	0 h	8.00	9.11	9.04	8.88	5.73
F8_1	0 h	<2	<2	<2	6.86	<4
	14 days	<2	<2	<2	<2	<2
	28 days	<1	<1	<1	<1	<1
F8_2	0 h	<2	3	<2	6.81	<4
	14 days	<2	<2	<2	<2	<2
	28 days	<1	<1	<1	<1	1
F8_3	0 h	<2	4.77	<2	6.93	<4
	14 days	<2	<2	<2	<2	<2
	28 days	<1	<1	<1	<1	1.69

## Data Availability

Data are unavailable due to privacy restrictions.
